# Editorial

**Published:** 1849-05

**Authors:** 


### THE MEDICAL EXAMINER.

### PHILADELPHIA, MAY, 1849.

Editors of the Medical Examiner.

Will you spare me a corner in your valuable Journal, to make known the final settlement, recently, of a case involving the legal rights of physicians to charge for professional services rendered the Commonwealth as witnesses in criminal cases? The establishment of this claim is a matter of interest and importance to the profession. About nine years since, Dr. J. M. Wallace and myself examined, at the instance of the Coroner, the body of a child who had died, as was alleged, from the effects of poison administered by a servant in the family. The chemical analysis of the contents of the primæ viæ was conducted at the expense of much time, labour, and material, by Dr. R. E. Rogers, then living in Philadelphia, and upon the trial we were all examined as medical witnesses, to prove the existence of the poison. Proper bills for the service, exclusive of ordinary witness-money, were rendered at the time to the prosecuting officer of the Court, and by him endorsed to the County Commissioners for settlement. Payment, however, was refused, not from any indisposition, it was said, to compensate us for the services, but simply from a supposed want of proper legal authority to do so. The claim, consequently, was pushed no further until a late decision of the Supreme Court, in a similar case, had settled the point, that professional service rendered at the instance of a proper legal officer, was entitled to special compensation by the County. In conformity with this decision, an appropriation was lately made for the settlement of our bills.

Hitherto, medical men have been subjected to much labour and vexation in medico-legal cases, without receiving any pecuniary compensation, the legal tribunals, like the public generally, expecting that physicians would of course always be willing in such cases to render their professional services gratuitously.

For the future, however, it should be understood that the law *must pay* when it needs a medical opinion in order to promote the ends of justice, and every one will see at once the indispensable necessity of such testimony in trials for murder charged to have been committed by means of poison. It is high time, we conceive, that the profession had taken a firm stand in defence of its just claims to remuneration, not only by courts of justice, but in other quarters also, where its charities are so liberally appealed to, more especially, too, as its members are liable to be mulcted in heavy damages upon charges of neglect merely; and we trust, therefore, that a *reform* in this, as well as in other matters, will not be indefinitely postponed.

Yours, &c.,

FRANCIS WEST.

*Philada.*, April 6, 1849.

[We endorse the above with all our hearts, more especially as we too have been sufferers in circumstances nearly similar. It is indeed high time that the medical profession were looking after their own interests in these matters, and in every instance where professional opinions and valuable time are demanded for the purpose of justice, that they should insist upon proper remuneration for their services. Medical witnesses are not only called upon to render time and learning to further the ends of justice, but are obliged often to submit to the impertinent badgering and cross-examinations of counsel, very frequently more for their own amusement and the display of their little smattering of medical knowledge, than for any positive advantage that may accrue to the case under trial. We hope, therefore, now that the precedent is established, that no medical witness will fail to claim and sue for proper remuneration for services rendered.—EDS.]

EDITORIAL CHANGES.

Drs. Drake and Colescott have retired from the editorial department of the Western Journal of Medicine and Surgery. It is now in the hands of Prof. Yandell, assisted by Dr. P. S. Bell, a former associate.

PROFESSORIAL CHANGES.

Dr. Drake has retired from the University of Louisville, in consequence of his removal to Cincinnati.

Drs. Bartlett and T. D. Mitchell have resigned their professorships in Transylvania University. Dr. H. M. Bullitt, of Louisville, has accepted the chair of Materia Medica vacated by the resignation of the latter gentleman.

Dr. Donné has retired from the chair of Anatomy in Memphis Medical College.

The Chair of Physiology, Pathology, and Pathological Anatomy in the University of Louisiana, made vacant by the death of Dr. Harrison, has been filled by the selection of Dr. Thomas Hunt, of New Orleans.

We also learn through the Boston Medical and Surgical Journal, that Geo. Hayward, M. D., has resigned the professorship of Surgery in Harvard University.

MEDICAL SOCIETY OF THE STATE OF PENNSYLVANIA.

[Through the kindness of Dr. Patterson, Recording Secretary of the Society, we have been favoured with a copy of the minutes of the first annual meeting, from which the following abstract was made.—Eds.]

The Society held its annual meeting at Reading, April 11th, 1849. Delegates were present from Philadelphia, Lancaster, Berks, Alleghany, Bucks, Chester, Montgomery, Lycoming and Schuylkill counties, in all thirty-six, including the associate members not re-appointed to this meeting. Dr. Samuel Humes, of Lancaster, presided, by whom an eloquent address was delivered at the opening of the session, in which he paid a merited tribute of praise to Dr. Edwards, M. C., for his labours in behalf of the profession. The address was ordered to be printed.

Among the resolutions offered at this meeting was the following:

"*Resolved*, That any charge which may be made against any member of this body, affecting his character as a regular practitioner of medicine, be referred to a committee of five members, with instructions to report thereon before the final adjournment of the Society."

Under this resolution, charges of irregularity in practice were preferred against a member of the Society, and a committee appointed to examine into them, who reported in substance, as follows: That they had held a conference with the suspected member, by whom they were assured "that he had no affinity with the homœopathists; that he has no belief in the truth of their notions; that he wishes to do nothing to promote the success of that class of irregular practitioners; that, although he has used medicines such as those employed by homœopathists, they were not used by him upon homœopathic principles; and that, though he has been in one instance present with a homœopathic practitioner, he has in no case held a consultation with them." Under these circumstances, the committee did not consider the charge substantiated. The report of the committee was accepted.

The following amendment to the Constitution, Art. 6, Sec. 1, was offered by Dr. Atlee:

"*Provided also*, That the members of the profession in the city and county of Philadelphia, may, if they deem it expedient, form three Societies within their limits, of which the College of Physicians, now existing, shall be one."

On motion of Dr. Worthington, the following amendment was added:

"*And provided also*, That in appointing their representation to the State Society, no person belonging to two or more Societies shall be counted in more than one." The amendment being accepted, the whole motion was carried.

A committee, consisting of Drs. Kittoe, Norris, and Patterson, was appointed to draft an address to the profession throughout the State, calling their attention to the importance of the State Society.

An abstract from the minutes of the Chester County Medical Society was read, instructing their delegates to urge upon the State Society the propriety of making some provision for the members of the profession and their families, when from sickness or accident they may be disqualified from attending to their professional duties. In relation to which Dr. Wood offered the following resolution:

*Resolved*, That a Medical Beneficial Fund be established by voluntary contribution, that the treasurer of this Society act as treasurer of said Fund, and that he invest the sum contributed to this fund, and the interest thereon, so often as it amounts to $100, in State or City loan.

On motion of Dr. Atlee, a committee of five was appointed to consider the above resolution, together with the subject of mutual assurance among physicians, with instruction to report at the next meeting.

Several resolutions in relation to the adoption of a plan of universal vaccination throughout the State; the appointment of a committee to collect information in relation to the late epidemic of small pox throughout the State; and the adoption by the Legislature of a law to secure the registration of births, marriages and deaths, were adopted by the Society, and committees appointed on each.

The whole proceedings evince great interest and enthusiasm on the part of the members, and manifest a healthy condition of the profession throughout the State.

The following officers were appointed for the next annual meeting to be held in Philadelphia on the third Wednesday of April, 1850.

*President*, Prof. SAMUEL JACKSON, Philadelphia; *Vice Presidents*, Drs. E. D. KITTOE, Lycoming, W. WORTHINGTON, Chester, C. H. MATTHEWS, Bucks County, GEO. HALBERSTADT, Schuylkill County; *Corresponding Secretary*, Dr. ISAAC HAYS, Philadelphia; *Recording Secretaries*, Dr. H. S. PATTERSON, Philadelphia, and Dr. GEORGE B. KERFOOT, Lancaster; *Treasurer*, Dr. GEORGE FOX, Philadelphia; *Censors*, 1st and 2d Districts, Drs. F. A. MUHLENBERG, Lancaster, G. W. NORRIS, Philadelphia, W. WORTHINGTON, Chester, J. S. CARPENTER, Schuylkill, H. CORSON, Montgomery; 3d and 4th Districts, Drs. T. WOOD, Lycoming, —— RANKIN, Lycoming, A. DAVIDSON, Lycoming, W. MCILVAINE, York, T. S. HALLER, York; 5th and 6th Districts, Drs. J. P. GAZZAM, W. ADDISON, G. D. BRUCE, J. BROOKS, R. M. MOWRY, Alleghany.

The following gentlemen were appointed delegates to the American Medical Association at the annual meeting in May next: Drs. S. HUMES, S. DUFFIELD, T. WOOD, J. S. CARPENTER, F. S. BURROWES.

---

### NATIONAL CONVENTION FOR REVISING THE PHARMACOPŒIA OF THE UNITED STATES.

The Convention for revising the Pharmacopœia, which met in Washington, in January, 1840, adopted the following resolutions:

"1. The President of this Convention shall, on the first day of May, 1849, issue a notice requesting the several *incorporated State Medical Societies*, the *incorporated Medical Colleges*, the *incorporated Colleges of Physicians and Surgeons*, and the *incorporated Colleges of Pharmacy*, throughout the United States, to elect a number of delegates, not exceeding three, to attend a general Convention to be held at Washington on the first Monday in May, 1850.

"2. The several incorporated bodies, thus addressed, shall also be requested by the President to submit the Pharmacopœia to a careful revision, and to transmit the result of their labours, through their delegates, or through any other channel, to the next Convention.

"3. The several medical and pharmaceutical bodies shall be further requested to transmit to the President of this Convention the names and residences of their respective delegates as soon as they shall have been appointed, a list of whom shall be published, under his authority, for the information of the medical public, in the newspapers and medical journals, in the month of March, 1850.

"4. In the event of the death, resignation, or inability to act of the President of the Convention, these duties shall devolve on the Vice President; and, should the Vice President also be prevented from serving, upon the Secretary, or the Assistant Secretary, the latter acting in the event of the inability of the former."

In compliance with the foregoing resolutions, the undersigned, having been informed by the President of the late Convention, Dr. Lewis Condiet, that he would be unable, from indisposition, to perform the duties assigned to him, gives notice to the several Medical and Pharmaceutical bodies enumerated in the first resolution, that a Convention for the revision of the National Pharmacopœia, will meet in the city of Washington, on the first Monday in May, 1850. The undersigned also requests of the several bodies referred to, that they will fulfil the wishes of the Convention, as set forth in the second resolution; and, further, that they will transmit to his address, on or before the first of March next, the names and residences of the delegates whom they may appoint, in order that a list of them may be published, as directed in the third resolution.

GEO. B. WOOD, M.D.
Vice President of the Convention of 1840.

*Philadelphia,* May 1st, 1849.

